# MLVA Subtyping of Genovar E *Chlamydia trachomatis* Individualizes the Swedish Variant and Anorectal Isolates from Men who Have Sex with Men

**DOI:** 10.1371/journal.pone.0031538

**Published:** 2012-02-21

**Authors:** Olivia Peuchant, Chloé Le Roy, Björn Herrmann, Maithé Clerc, Cécile Bébéar, Bertille de Barbeyrac

**Affiliations:** 1 Université de Bordeaux, USC Mycoplasmal and Chlamydial Infections in Humans, French National Reference Centre for Chlamydial Infections, Bordeaux, France; 2 INRA, USC Mycoplasmal and Chlamydial Infections in Humans, French National Reference Centre for Chlamydial Infections, Bordeaux, France; 3 CHU de Bordeaux, Laboratoire de Bactériologie, Bordeaux, France; 4 Section of Clinical Bacteriology, Department of Medical Sciences, Uppsala University, Uppsala, Sweden; St. Petersburg Pasteur Institute, Russian Federation

## Abstract

This study describes a new multilocus variable number tandem-repeat (VNTR) analysis (MLVA) typing system for the discrimination of *Chlamydia trachomatis* genovar D to K isolates or specimens. We focused our MLVA scheme on genovar E which predominates in most populations worldwide. This system does not require culture and therefore can be performed directly on DNA extracted from positive clinical specimens. Our method was based on GeneScan analysis of five VNTR loci labelled with fluorescent dyes by multiplex PCR and capillary electrophoresis. This MLVA, called MLVA-5, was applied to a collection of 220 genovar E and 94 non-E genovar *C. trachomatis* isolates and specimens obtained from 251 patients and resulted in 38 MLVA-5 types. The genetic stability of the MLVA-5 scheme was assessed for results obtained both *in vitro* by serial passage culturing and *in vivo* using concomitant and sequential isolates and specimens. All anorectal genovar E isolates from men who have sex with men exhibited the same MLVA-5 type, suggesting clonal spread. In the same way, we confirmed the clonal origin of the Swedish new variant of *C. trachomatis*. The MLVA-5 assay was compared to three other molecular typing methods, *ompA* gene sequencing, multilocus sequence typing (MLST) and a previous MLVA method called MLVA-3, on 43 genovar E isolates. The discriminatory index was 0.913 for MLVA-5, 0.860 for MLST and 0.622 for MLVA-3. Among all of these genotyping methods, MLVA-5 displayed the highest discriminatory power and does not require a time-consuming sequencing step. The results indicate that MLVA-5 enables high-resolution molecular epidemiological characterisation of *C. trachomatis* genovars D to K infections directly from specimens.

## Introduction

*Chlamydia trachomatis* is the bacterium most commonly responsible for sexually transmitted infections. Most of these infections are asymptomatic and, if not treated, can lead to severe complications and sequelae. *C. trachomatis* is currently divided into 19 genovars, according to the type of its major outer membrane protein (MOMP) epitopes [1]. Genovars D to K are associated with urogenital infections. According to epidemiological studies, genovar E is the most prevalent, responsible more than 40% of urogenital *C. trachomatis* infections in the heterosexual population [2], [3], [4], [5]. This genovar is also involved in 10% of the men who have sex with men (MSM) with proctitis in France [6]. Genotyping may reveal transmission patterns in sexual networks, play a role in cases of sexual abuse or assaults, help to determine whether infections are persistent or new, and aid monitoring of the emergence of specific clones. Examples of emerging clones include the lymphogranuloma venereum (LGV) proctitis outbreak caused by the L2b strain among MSM and more recently, the clonal spread of the Swedish new variant (nvCT) characterised by a 377-bp deletion in the cryptic plasmid [3], [7], [8].

Early techniques for molecular typing of *C. trachomatis* were developed based on the *ompA* gene encoding MOMP, known as *ompA* genotyping [9]. This approach allows classification in genovars corresponding to serovars, using polymerase chain reaction (PCR) - restriction fragment length polymorphism (RFLP), *ompA* gene sequencing and more recently real-time PCR and microsphere suspension arrays [10]. Other typing methods targeting all or parts of the genome have also been developed. Some methods such as pulse field gel electrophoresis (PFGE), random amplification of polymorphic DNA (RAPD) or amplified fragment length polymorphism (AFLP) are time-consuming or present problems in reproducibility and discrimination [10]. Other techniques more reproducible and associated with a high level of discrimination seem more promising. These include multilocus sequence typing (MLST) [11], [12], [13] and a four-loci method combining *ompA* sequencing and a multilocus variable number tandem-repeat (VNTR) analysis (MLVA) method that relies on the number of single nucleotide repeats within three loci, called MLVA-3 in this study [8]. Among these many methods, the MLST technique developed by Klint *et al.* and the MLVA-3 method demonstrate a good degree of resolution, which is needed for isolate discrimination and outbreak investigation [14], [15].

The aim of the present study was to set up a new MLVA scheme, automated and without a sequencing step, for *C. trachomatis* urogenital genovars typing. This method was developed on genovar E isolates and then applied on a wide range of *C. trachomatis* genovar D to K isolates and specimens. To evaluate the discriminatory power of this technique among isolates from the same genovar, we compared it to those obtained with *ompA* sequencing, the MLST technique of Klint *et al.* and the MLVA-3 method on 43 genovar E isolates.

## Materials and Methods

### Ethics statement

The present project is in compliance with the Helsinki Declaration (Ethical Principles for Medical Research Involving Human Subjects). The study was conducted in accordance with the guidelines of the “Direction de la Recherche Clinique et de l'Innovation” of the ethical board Bordeaux University hospital, Bordeaux, France. All patient data were anonymously reported, with no possibility of connecting the isolates to individual patients.

French specimens were collected as part of routine patient management without any additional sampling, and patients provided no objection for their samples to be used. According to the article L1123-7 of the French code of Public Health, this study did not need to be examined by the ethical committee “Comité pour la Protection des Personnes” and allowed the exemption of patient's written or oral informed consent. Written informed consent was obtained from French patients to collect sexual bevahiour information following the authorization of the ethical committee “Comité Consultatif pour le Traitement de l'Information en matière de Recherche dans le domaine de la Santé” (No. 10.362).

Swedish specimens from Uppsala University Hospital were collected as part of routine diagnostics and were anonymised. Informed consent was not needed for use of microbiological samples in quality assurance of diagnostic methods according to the Biobanks in Medical Care Act (2002:297). Collection and use of anonymised samples was approved by the Regional Ethical Review Board in Uppsala, Sweden (Dnr 2007/312).

### Clinical isolates and specimens

- **Genovar E.** The E/Bour reference strain, 145 French *C. trachomatis* genovar E clinical isolates, and 74 French and Swedish genovar E positive-*C. trachomatis* clinical specimens from 166 patients were used in this study (Table S1). All of the French isolates and specimens were collected between 1987 and 2009 at the French national reference centre for chlamydial infections (Bordeaux, France). These isolates and specimens were divided into six groups (Table S1). Group I included 103 French urogenital clinical isolates and the corresponding specimens for 12 of them. In Group II, concomitant urogenital isolates and specimens collected from 17 French patients were analysed. Group III consisted in French urogenital sequential isolates and specimens collected from 12 patients. Group IV consisted in French *C. trachomatis*-positive specimens collected before and after antibiotic treatment from five patients with an interval of 8–10 weeks between the two specimen collections. Group V was composed of the unique nvCt isolate identified in France [16] and 20 Swedish specimens collected in 2009 at the Uppsala University Hospital (Uppsala, Sweden) and containing the recently described nvCT strain [3]. Finally, Group VI contained 10 male and one female anorectal isolates and specimens collected in France from 2005 to 2010.

The *C. trachomatis* isolates were cultured on McCoy cells as previously described [9].

- **Non-E genovar.** Reference strains D/UW-3/Cx, Da/TW-448/Cx, F/IC-Cal-3, G/UW-57/Cx, H/UW-43/Cx, I/UW-12/Ur, Ia/UW-202/Cx, J/UW-36/Cx and K/UW-31/Cx were analysed. Then, we genotyped 85 *C. trachomatis*-positive specimens (54 urogenital and 31 anorectal) belonging to genovars D, Da, F, G, H, I, Ia, J and K (Table S2). Specimens were collected between 1994 and 2010 at the French national reference centre for chlamydial infections (Bordeaux, France).

### DNA isolation

DNA was extracted from clinical specimens or bacterial cell cultures using the MagNA Pure LC DNA Isolation Kit I (Roche, Meylan, France) according to the manufacturer's instructions.

### MLVA-5 analysis

VNTR markers were identified in the sequenced genomes of *C. trachomatis* A/HAR-13 [17], D/UW-3/Cx [18], L2 434/Bu, L2b/UCH-1/proctitis [19] and E/Sweden2 [20] by the Microorganisms Tandem Repeats database and the Tandem Repeats Finder program which is available at http://minisatellites.u-psud.fr. To improve polymorphic tandem repeat (TR) identification, loci were chosen on the basis of matches of at least 80% between the DNA sequences of the repeat units. A total of 202 TRs were selected and designated by the letters Ct followed by a number corresponding to the position on the *C. trachomatis* D/UW-3/Cx genome. To screen for variability in the number of TRs, PCR primers targeting the regions flanking TR loci were designed and tested on a set of 19 genovar E *C. trachomatis* isolates, including the E/Bour reference strain, eight cervix isolates, five male urethral isolates, four anorectal isolates and one isolate from peritoneal fluid, collected from 1987 to 2008 from different geographical areas. Each locus was amplified individually and analysed by conventional agarose gel electrophoresis. To confirm that length polymorphisms were the result of repeat copy number variations, the PCR products were purified using Wizard PCR Preps DNA Purification System (Promega, Charbonnières-les-Bains, France) and sequenced using fluorescence-labelled dideoxynucleotide technology, according to the manufacturer's instructions (Applied Biosystems, Courtaboeuf, France). Based on this approach, five VNTR markers were selected for full assessment.

The five VNTR markers ultimately selected for MLVA-5 were multiplexed in two solutions: a triplex PCR was used to amplify the markers Ct-51, Ct-531 and Ct-1025 and a duplex PCR was used to amplify the markers Ct-719 and Ct-1035. Amplifications were performed with a Mastercycler ep gradient S apparatus (Eppendorf, Hamburg, Germany) in a final volume of 25 µl. The reaction mixtures contained 1× Qiagen PCR buffer with 1.5 mM MgCl_2_, 0.2 mM deoxynucleotide triphosphate, 3 mM MgCl_2_, 0.625 U of Hot Start *Taq* DNA polymerase (Qiagen, Hilden, Germany), 0.125 µM of each primer and 1 µl of template DNA for clinical isolates or 2.5 µl of template DNA for clinical specimens. Forward primers were fluorescently labelled at the 5′ end using 4,4,7,2′,4′,5′,7′-hexachloro-6-carboxy-fluorescein (HEX), 6-carboxyfluorescein (FAM; Eurogentec, Angers, France) or NED (2′-chloro-5′-fluoro-7′,8′-fused phenyl-1,4-dichloro-6-carboxyfluorescein; Applied Biosystems), depending on the locus to be amplified (Table 1). Both multiplex solutions were run under the same cycling conditions: 95°C for 15 min followed by 25 cycles of 95°C for 1 min, 60°C for 1 min and 72°C for 1 min and a final extension at 72°C for 10 min. For clinical specimens, the cycling conditions were similar, except that amplification required 40 cycles. Prior to GeneScan analysis, 0.3 µl of GeneScan ROX 500 size standard (Applied Biosystems) was added to 1 µl of each PCR product. After heat denaturation for 5 min at 95°C, the fragments were separated using an ABI 3130 Genetic Analyzer (Applied Biosystems). The GeneScan data were subsequently analysed using GeneMapper software (version 3.7; Applied Biosystems) to perform sizing and to calculate the number of repeats in the PCR fragments. Each locus was identified according to colour fluorescence. An allele number string, based on the number of repeats at each locus, was assigned to all isolates. The calculated numbers of repeats were imported in BioNumerics (version 6.1; Applied Maths) for further cluster analysis.

**Table 1:** Oligonucleotide primers used for MLVA-5.

Primer name^a^	Dye sequence (5′→3′)
Ct-51-fwd………………	HEX-TGGGAGTACAAAAGAAATGCAG
Ct-51-rev………………	GAAGCAGCTACACGTCCACA
Ct-531-fwd…………….	NED-CCTCATCAAGCGATCATATCC
Ct-531-rev………………	GCCGAATACACCCTCGTTAC
Ct-719-fwd…………….	HEX-CTCGCAACCCCAGAGTCG
Ct-719-rev……………‥	GTGTTTTGTTATGTGTGGTAGGCT
Ct-1025-fwd……………	6-FAM-CCATCGTCGAGCCATATCTT
Ct-1025-rev…………….	TCTTCCGTCCCCTCCTCTT
Ct-1035-fwd……………	6-FAM-CCGGAGCAGGCGAAGTG
Ct-1035-rev…………….	CCTTTCCGAGCATCACTAACTG

a fwd, forward primer; rev, reverse primer.

### Other typing techniques for *C. trachomatis*

Three methods (*ompA* sequencing, MLST and MLVA-3) relying on sequencing of selected genes were performed on 43 genovar E isolates (in bold in Table S1) belonging to 15 MLVA-5 types. This group included all anorectal isolates, the nvCT and extragenital isolates, isolates with a unique or infrequent MLVA-5 type, isolates belonging to the three main MLVA-5 types and concomitant or sequential isolates from the same patient.

For *ompA* sequencing, a 1085-bp fragment of this gene was amplified by PCR using CT1 and CT5 primers [9]. Sequence data of the new allelic variant is avalaible under GenBank accession number JN192145.

For the MLST method, five regions of the *C. trachomatis* genome (*hctB*, CT058, CT144, CT172 and *pbpB*) were amplified as described by Klint *et al.* [12]. The PCR products were purified and sequenced as described above. Sequences were submitted to the Uppsala *C. trachomatis* MLST database (http://mlstdb.bmc.uu.se) and compared with previously described allele variants to determine the sequence type (ST) of the isolates. Concerning the new allelic variants and ST assignment, we used the numbering system developed originally in 2007 by Klint *et al.*. We described 11 new allelic variants: *hctB*-54 (GenBank accession number HQ37008), *hctB*-55 (GenBank accession number HQ37009), *hctB*-56 (GenBank accession number JN192138), *hctB*-57 (GenBank accession number JN192137), *hctB*-58 (GenBank accession number JN192139), *hctB*-59 (GenBank accession number JN192140), CT058-46 (GenBank accession number JN192144), CT058-47 (GenBank accession number JN192143), CT172-17 (GenBank accession number HQ873007), *pbpB*-54 (GenBank accession number JN192141) and *pbpB*-55 (GenBank accession number JN192142). We described 12 new STs, ST 222 to 233 (Table 2).

**Table 2:** Description of the 12 new MLST sequences types.

*hctB*	CT058	CT144	CT172	*pbpB*	ST
1	2	6	**17**	2	222
**59** a	2	7	2	**54**	223
**58**	19	7	2	1	224
21	2	7	2	1	225
**56**	19	7	2	1	226
**57**	2	7	2	1	227
**54**	**46**	7	2	1	228
1	19	7	**17**	1	229
5	19	7	2	1	230
**55**	19	7	2	1	231
1	2	6	2	1	232
1	2	6	2	**55**	233
na^b^	**47**	6	2	2	nd^c^

a new allelic variants are indicated in bold.

b na: no amplification.

c nd: not determined.

For the MLVA-3 method, three loci (CT1291, CT1299 and CT1335) were amplified as described by Perdersen *et al.* [8]. The assignment of the MLVA-3 type was carried out according to the rules described by Pedersen *et al.* [8].

### Data analysis

The polymorphism index of individual or combined VNTRs was calculated using the Hunter-Gaston discriminatory index (HGDI) [21]. For MLVA-5, the HGDI was calculated on 146 genovar E and 94 non-E genovar isolates and specimens that were epidemiologically unrelated. Data obtained from MLVA-5 for genovar E isolates and specimens were converted into character data sets and analysed using BioNumerics software. A minimum spanning tree (MST) was generated to visualise of the relationships between large numbers of isolates in a single compact image. The MST was created based on the categorical coefficient and a priority rule consisting of the highest number of single-locus variants.

For the comparison of the molecular typing methods, the HGDI if each technique was calculated on 38 genovar E isolates that were epidemiologically unrelated (for concomitant or sequential isolates from the same patient, only the first isolate was included). Data obtained from each method were analysed using BioNumerics software. A MLVA-5 dendrogram including the results of other typing methods was constructed using the categorical coefficient and the unweighted-pair group method using arithmetic averages (UPGMA) analysis.

## Results

### Identification of VNTRs for MLVA typing

Amplification of 202 loci from the E/Bour reference strain and 18 genovar E isolates showed that only five loci were polymorphic with different allele sizes. A total of 195 markers were monomorphic, and two markers were not useful for VNTR analysis since the sequence variability observed between isolates was not related to a variation in repeat number but to insertions/deletions. The five VNTRs were distributed around the genome from nucleotide positions 51543 to 1035380 in the *C. trachomatis* D/UW-3/Cx reference strain (Table 3). The PCR products ranged in size from 151–604 bp in the *C. trachomatis* E/Bour reference strain. Four of the five VNTRs were located in open reading frames (ORFs). The markers Ct-51, Ct-531 and Ct-1035 were located in the *hctB* gene encoding the histone-like protein Hc2, the *tarp* gene encoding a translocated actin-recruiting protein and the *pmpH* gene encoding a polymorphic outer membrane protein, respectively. The marker Ct-1025 was located in an ORF encoding a hypothetical protein. The fifth VNTR, Ct-719, was in an intergenic region. Characteristics of each VNTR marker are presented in Table 3. The sizes of the unit repeats ranged from 3 bp for Ct-719 and Ct-1025 to 150 bp for Ct-531. Sequencing of the PCR products of different sizes at each of the five loci from each of the 19 screening isolates confirmed the sizes and sequences of the individual VNTR loci.

**Table 3:** Characteristics of the five VNTR markers.

Name	Nucleotide position^a^ (bp)	Locus (protein no. in the genome sequence)	Repeat size(bp)	% identity between VNTRs	Locus size(bp)
Ct-51	51543	*hctB* gene (CT046)	108	91	307–604
Ct-531	531356	*tarp* gene (CT456)	150	98	294–594
Ct-719	719983	Intergenic	3	100	206–209
Ct-1025	1025053	Hypothetical protein (CT868)	3	94	244–253
Ct-1035	1035358	*pmpH* gene (CT872)	6	83	144–150

a Position (5′ end) on the *C. trachomatis* D/UW-3/Cx genome sequence.

The use of fluorescently labelled primers in a triplex PCR associating Ct-51, Ct-531 and Ct-1025 and a duplex PCR, including Ct-719 and Ct-1035 and adapted to capillary electrophoresis, facilitated the interpretation of the results in contrast to agarose gel electrophoresis. Using GeneMapper software, all loci were clearly identified on electropherograms according to their size ranges and colours, and the amplicon sizes allowed the determination of the repeat number.

### Specificity and stability of the MLVA-5 scheme

The specificity of MLVA-5 assay was investigated by conducting PCR on 15 *C. trachomatis*-negative clinical specimens and DNA from the following bacterial species: *Escherichia coli*, *Enterococcus faecalis*, *Klebsiella pneumoniae*, *Gardnerella vaginalis*, *Candida albicans*, *Lactobacillus* spp., *C. pneumoniae*, *Mycoplasma hominis*, *M. genitalium*, *Ureaplasma* spp., *Neisseria gonorrhoeae*, *Staphylococcus aureus* and *S. epidermidis*. No amplification was observed for all of the non-*C. trachomatis* isolates and specimens, thereby confirming the specificity of the assay.

The E/Bour reference strain and four clinical *C. trachomatis* genovar E isolates (I_E_783, I_E_802, I_E_874 and I_E_SL52) belonging to four different MLVA-5 types were used in the stability study. Each isolate was serially passaged 10 times on McCoy cells to determine the stability of each locus before and after passaging. The DNA was subsequently extracted and double-strand sequenced to identify the number of repeats in each locus. Analysis of the five isolates resulted in identical MLVA-5 type for all markers.

### MLVA-5 typing of clinical isolates and specimens

The five VNTRs were efficiently amplified for all 314 *C. trachomatis* isolates and specimens tested, including those for which culture could not be achieved. Analysis of the results revealed 38 MLVA-5 types, from 01 to 38 (Table 4). The HGDI of the MLVA-5 scheme was 0.919.

#### Genovar E clinical isolates and specimens

**Table 4:** Number of repeat units of the five VNTR markers in 220 genovar E and 94 non-E genovar *C. trachomatis* isolates and clinical specimens.

MLVA-5 type	No. of repeats at the following VNTR loci					No. of isolates by MLVA-5 type	
	Ct-719	Ct-1035	Ct-51	Ct-531	Ct-1025	genovar E	non-E genovar
01	1	4	4.5	2	3	6	0
02	2	4	4.5	2	3	44	0
03	2	4	3.5	2	3	2	0
04	2	4	4.5	1	3	1	0
05	2	4	4.5	2	6	3	0
06	2	4	2.5	2	6	2	0
07	2	3	2.5	2	3	1	0
08	1	4	3.5	2	3	4	6
09	1	4	3.5	3	3	2	12
10	1	3	4.2	2	6	4	0
11	1	3	3.5	2	6	40	3
12	1	3	4.5	1	6	3	0
13	1	3	4.5	2	6	55	0
14	1	3	2.5	2	6	21	0
15	1	3	2.5	3	6	2	1
16	1	4	2.5	2	6	1	0
17	1	4	3.5	2	6	1	17
18	1	4	4.5	2	6	1	0
19	1	3	4.5	2	3	8	0
20	2	3	4.5	2	3	1	0
21	1	3	4.5	2	4	10	0
22	1	4	4.5	2	4	2	0
23	2	4	3.5	2	6	2	0
24	1	3	3.7	2	6	2	0
25	2	4	3.5	3	3	2	0
26	1	4	3.5	3	4	0	10
27	1	4	3.5	3	6	0	9
28	1	4	3.5	2	4	0	4
29	1	4	3.5	1	4	0	1
30	1	4	2.5	3	4	0	3
31	1	4	2.5	3	3	0	2
32	1	3	3.5	3	6	0	18
33	1	4	2.5	3	6	0	2
34	1	4	2.5	1	3	0	1
35	1	4	2.5	2	4	0	1
36	1	4	3	3	4	0	2
37	1	4	3	3	3	0	1
38	1	4	3.5	1	3	0	1

**Figure 1:**
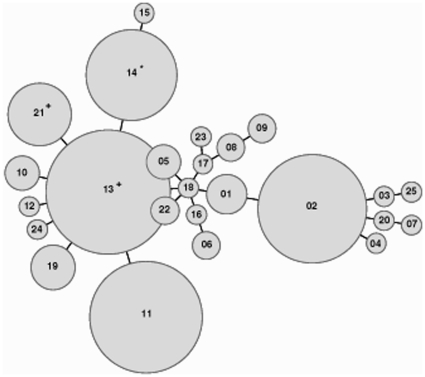
MST of the MLVA-5 types of genovar E *C. trachomatis* isolates and positive specimens. Each circle denotes a particular MLVA-5 type. The size of the circle is proportional to the number of isolates or specimens belonging to the indicated MLVA-5 genotype. The distance between neighbouring genotypes is expressed as the number of allelic changes and is outlined by short bold lines for one change. No cluster is emphasised as shown by the distance, equal to one, between neighbouring genotypes. **^+^** MLVA-5 types encompassing anorectal isolates and specimens. ^★^ MLVA-5 type containing nvCT isolate and specimens.

Genovar E isolates and specimens were of 25 MLVA-5 types, from 01 to 25 (Table S1). Three MLVA-5 types, 02, 11 and 13, included 63% of all genovar E isolates and specimens tested (Figure 1). In 12 cases (5%), one unique MLVA-5 type was observed for a single patient. The size variation of the amplicons was in exact multiples of the repeats, except for the marker Ct-51 for which partial repeats were found (Table 4). The marker Ct-51 showed five different allele sizes, with repeat copy numbers ranging from 2.5 to 4.5. The markers Ct-531 and Ct-1025 displayed three different allele sizes, and the markers Ct-719 and Ct-1035 resulted in two different-sized PCR products. The marker Ct-531 was the most homogeneous with 140 isolates or positive specimens harbouring two repeat units of 150 bp. This was reflected by the diversity index of each VNTR as estimated by the HGDI, yielding a value of 0.546 for the most discriminatory marker (Ct-1025) and a value of 0.080 for Ct-531, which is the least discriminatory marker. The overall discriminatory index for MLVA-5 was 0.839.

The reliability of MLVA-5 was affirmed by the results obtained from typing direct clinical specimens and that of the corresponding isolate (Group I) as they all resulted in the same MLVA-5 type per patient (Table S1).

In Group II, no intra-individual variation of the MLVA-5 type was found for the 17 patients with concomitant isolates or specimens. In Group III, sequential isolates and specimens from each of the 11 patients revealed an identical MLVA-5 type suggesting a persistent *C. trachomatis* infection or a possible reinfection by an untreated partner (Table S1). For one patient, three concomitant isolates from the female locations cervix, peritoneal fluid and endometrium obtained on the day of testing shared the MLVA-5 type 02, while the cervix isolate obtained three years later belonged to the MLVA-5 type 13, suggesting a new *C. trachomatis* infection (Table S1).

For five patients (Group IV) who had been treated with single-dose azithromycin and in whom *C. trachomatis* could subsequently be detected, all patients showed an identical MLVA-5 type before and after treatment.

In Group V comprising the nvCT isolate obtained in France and 20 Swedish nvCT specimens, all displayed the unique MLVA-5 type 14, thereby confirming a clonal origin (Figure 1). In Group VI, of the 11 anorectal isolates and specimens, nine were of the MLVA-5 type 21 and these were all from MSM, while two other anorectal specimens, derived from a woman and a bisexual man, were of the MLVA-5 type 13. It should be noted that one additional isolate obtained from the male urethra displayed the MLVA-5 type 21 (Table S1).

Finally, the genetic relationships investigations showed no obvious link between the MLVA-5 type and the isolate or specimen year of collection or patient

#### Non-E genovar specimens

Non-E genovar specimens were of 18 MLVA-5 types, including 13 new MLVA-5 types (26 to 38) compared to the genovar E isolates and specimens (Table 4). Seven new MLVA-5 types were only constituted of anorectal specimens and four of them were of only a single specimen (Table S2). Three MLVA-5 types, 09, 17 and 32 included half of all non-E genovar specimens analysed. Interestingly, no size variation was observed for the Ct-719, with only one repetition for all specimens tested. The Ct-51 displayed only three different allele sizes, with repeat copy numbers ranging from 2.5 to 3.5. As described for genovar E, the Ct-531 and the Ct-1025 displayed three different sizes and the Ct-1035 two different-sized PCR products. The Ct-1025 was also the most discriminating marker with an HGDI equal to 0.614.

### Comparison of MLVA-5, *ompA* sequencing, MLST and MLVA-3 for molecular typing of genovar E *C. trachomatis* isolates

Forty-three genovar E isolates were included in an evaluation where MLVA-5 was compared to *ompA* sequencing, the MLST developed by Klint *et al.* and MLVA-3 [8], [12].

The MLVA-5 differentiated into 15 genotypes. *OmpA* sequencing resulted in two genotypes where all isolates except one displayed identical sequences compared to that of the E/Bour reference strain. The I_E_828 isolate harboured a silent A→G mutation at nucleotide position 420 in the constant domain C2 of the *ompA* gene.

**Figure 2:**
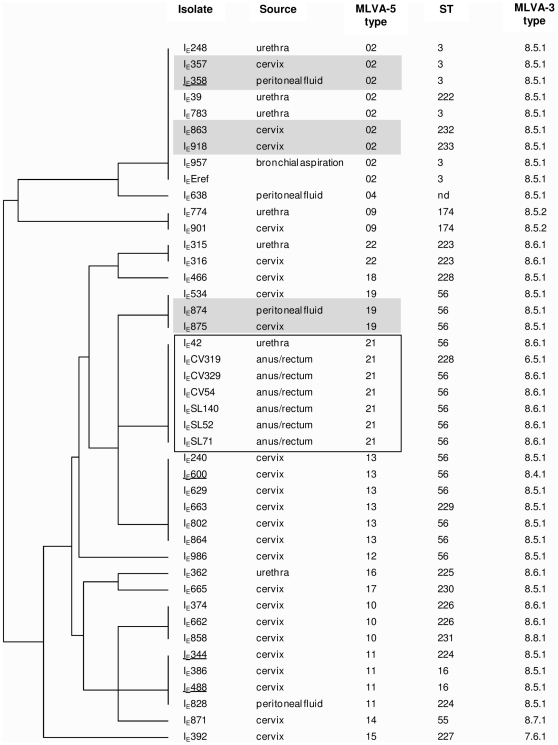
Clustering dendrogram of 43 genovar E *C. trachomatis* isolates typed by four molecular methods. The order in which the isolates are listed, from top to bottom, is based on the MLVA-5 dendrogram (left side of the figure). The dendrogram was constructed using a categorical coefficient and UPGMA clustering. Concomitant and sequential isolates from the same patients are highlighted in grey and are underlined, respectively. The MLVA-5 type 21, which included all anorectal isolates, is outlined in black. nd, not determined.

MLST analysis resulted in 17 STs. Complete MLST profiles were obtained for 42 out of the 43 isolates tested, as the *hctB* gene of one isolate could not be amplified. We identified six new allelic variants for the *hctB* gene, two for the CT058 and *pbpB* genes and one for the CT172 gene. MLST analysis resulted in the description of 12 new allelic STs, eight of which corresponded to only one isolate (Table 2). One third of the isolates were of ST-56 (Figure 2).

Using MLVA-3, eight MLVA-3 types were assigned. More than two thirds of the isolates were of the two MLVA-3 types 8.5.1 (25 isolates) and 8.6.1 (11 isolates) (Figure 2). We identified one new MLVA-3 type, 7.6.1, for the I_E_392 isolate.

All anorectal isolates shared the MLVA-5 type 21, ST-56 and MLVA-3 type 8.6.1 except for the I_E_CV319 isolate (Figure 2). The latter harboured the MLVA-5 type 21 and a new ST, ST-228 (as well as the cervical isolate I_E_466), and the unique MLVA-3 type 6.5.1, already described for urogenital isolates [14]. Although the MLVA-5 type 21 was only found among all anorectal isolates except one, ST-56 and MLVA-3 type 8.6.1 were commonly detected among urogenital isolates (10 and six urogenital isolates, respectively). Therefore, MLVA-5 appears to be more efficient at discrimination of clusters.

One patient probably harboured a new *C. trachomatis* infection three years after the initial visit because sequential isolates I_E_358 and I_E_600 displayed the different MLVA-5 types 02 and 13, respectively, as well as distinct STs and MLVA-3 types. In contrast, the concomitant isolates I_E_874 and I_E_875, I_E_357 and I_E_358 shared the same *Chlamydia* genotype regardless of the molecular typing method used (Fig. 2).

The HGDI, calculated on 38 isolates that were epidemiologically unrelated, was 0.913 for MLVA-5, 0.860 for MLST and 0.622 for MLVA-3 (Table 5). The congruence between MLVA-5 and MLST was 60.3%, while it was lower (22.5%) between MLVA-5 and MLVA-3. An identical HGDI of 0.949, with the same number of 20 different genotypes described, was found when MLVA-5 and MLST or MLVA-5, MLST and MLVA-3 were combined into one analysis (Table 5).

**Table 5:** Discriminatory index for the molecular typing methods, single or combined, on genovar E *C. trachomatis* isolates.

Method	HGDI^a^
MLVA-5	0.913
MLST	0.860
MLVA-3	0.622
MLVA-5+MLVA-3	0.925
MLST+MLVA-3	0.929
MLVA-5+MLST	0.949
MLVA-5+MLST+MLVA-3	0.949

a HGDI, Hunter-Gaston discriminatory index. The HGDI was calculated on 38 isolates that were epidemiologically unrelated.

## Discussion

In this study, we present a new MLVA-based molecular typing system for the discrimination of genovar E and other genovar D to K of *C. trachomatis* strains. MLVA-5 is a reproducible and fast technique that does not require a sequencing step and can be standardised, thereby facilitating large-scale molecular epidemiological investigations. The application of multiplex PCR and capillary electrophoresis on a genetic analyzer enables a high-throughput analysis and allows easier interpretation of results in contrast to agarose gel electrophoresis, particularly for VNTRs with a small number of repeat units.

*C. trachomatis* displays remarkable genome sequence similarity and synteny, considering the different diseases and tissue tropism exhibited by the different genovars. Of 202 TR loci identified, only five were polymorphic on genovar E isolates, confirming the high genetic homogeneity of this species. In comparison, eight VNTRs were found in *C. psittaci* among 20 TRs and five VNTRs were identified among 34 TRs for *C. abortus* [22], [23].

The marker Ct-51, located in the *hctB* gene, showed six different allele sizes with a repeat copy number ranging from 2.5 to 4.5. This gene was one of the targets of the MLST technique developed by Klint *et al..* All of the nvCT isolates displayed the allelic variant hctB-21 with MLST and a 2.5 repeat for the marker Ct-51 with MLVA-5. Other genovar E isolates shared one of these two allelic variants, although they did not have the plasmid deletion. The marker Ct-531, located in the *tarp* gene, was the least discriminatory of the VNTRs identified for the genovar E. These results confirm the data of Lutter *et al.*, who showed that among 14 urogenital genotype E clinical isolates collected from different areas, 12 had Tarp sequences identical to that of the E/Bour reference strain [24]. However, the marker Ct-531 allowed distinction of the nvCT isolates from the other isolates harbouring identical allelic profiles on the four other VNTRs. Although the Ct-719 displayed two allele sizes among genovar E isolates, this marker was monomorphic among the urogenital non-E genovar specimens analysed. However, more specimens need to be tested to confirm these results.

The stability of the method was assessed *in vitro* by ten-passage culturing for five isolates and *in vivo* using sequential isolates and specimens from 11 patients on a time interval of 1–28 months. Moreover, in 17 cases, concomitant specimens or isolates harboured identical MLVA-5 types. Taken together, both the *in vitro* and the *in vivo* data showed no MLVA-5 type variation over time or within individuals, implying that our MLVA-5 scheme is genetically stable for short-term applications.

In our study, nine of the 11 anorectal *C. trachomatis* genovar E isolates displayed the same unique MLVA-5 type 21. All of these patients were MSM and were mainly from the same region in France, suggesting clonal spread of a strain. Interestingly, the available genome of the E/150 rectal isolate shared the MLVA-5 type 21 [25]. Unfortunately, no information about the sexual behaviour and geographical origin of the patient from whom the E/150 strain was isolated was available. Using whole-genome sequencing along with comparative genomics, Jeffrey *et al.* showed that there were only 1130 substitutions and 54 insertions or deletions between the E/150 male rectal and the E/11023 cervical genovar E isolates [25]. In their work, the authors identified three ORFs that were statistically correlated with rectal tropism only in genovar G, while none of these ORFs were statistically associated with rectal tropism in genovar E isolates. We genotyped six anorectal isolates from MSM using MLST and MLVA-3. All of the isolates except one belonged to ST-56 and MLVA-3 type 8.6.1. These two genotypes were associated with both anorectal and urogenital isolates, while the MLVA-5 type 21 was only associated with all anorectal isolates except one. Indeed, one male urethral isolate displayed the MLVA-5 type 21, but the sexual behaviour of the patient was unknown. The association of a unique MLVA-5 type with anorectal genovar E specimens from MSM must be confirmed by further analysis of additional specimens. In contrast, the 31 anorectal non-E genovar specimens were distributed among 15 MLVA-5 types and there was no relation between MSM anorectal specimens tested and a MLVA-5 type.

Our results confirmed a clonal spread of the nvCT, in accordance with other reports [3], [8]. The nvCT specimens tested in our study were previously genotyped by MLST, and all of them displayed the same unique MLST profile ST-55 and an identical *ompA* sequence to that of the E/Bour reference strain [3]. All of these specimens also displayed the same unique MLVA-5 type 14.

Recently, using *ompA* genotyping, it was shown that repeated *C. trachomatis* infections in adolescent women were due to reinfection in 54% of the cases, treatment failure in 14% of the cases and persistence without documented treatment in 2% of the cases [26]. In our study, we obtained, *C. trachomatis*-positive specimens from five patients before and after treatment and these were for each patient of an identical MLVA-5 type, suggesting treatment failure and persistence of infection. However, we could not exclude recontamination as no information on partner treatment was available.

In several studies, the molecular epidemiology of chlamydial infections was based on analysis of the *ompA* gene. However, to discriminate within genotypes, a single marker is inadequate because of the risk of recombination events [5], [11]. Therefore, more discriminating molecular techniques are needed [8], [11], [12], [13]. Klint *et al.* developed an MLST scheme targeting the five most variable regions of the chlamydial genome, while Pedersen *et al.* developed an MLVA method based on single nucleotide repeats, which are a type of VNTR, within three loci combined to *ompA* sequencing [8], [12]. MLST and MLVA-3 are time-consuming techniques that require a sequencing step. In our work, we compared MLVA-5 to *ompA* sequencing, MLST and MLVA-3 for 43 genovar E isolates. Results from *ompA* sequencing confirmed a high level of conservation of genovar E isolates [2], [5], [12], [27]. Using MLST, 42 isolates exhibited17 STs, of which 12 were new. For one isolate, an ST could not be assigned because we failed to amplify one target region. Using MLVA-3, all isolates were amplified, and we described only eight MLVA-3 types of which one was new. It should be noted that it is difficult to interpret the MLVA-3 results, as more than 11 single nucleotide repeats can be observed at one single locus, associated with the strong probability that the DNA polymerase, routinely used in PCR, may have generated sequence errors [14], [28]. Furthermore, the stability of this technique has not been fully assessed [8]. Our data were in agreement with those reported by Bom *et al.* who observed that for 13 genovar E specimens, the number of STs described (eight) was higher than the number of MLVA-3 types (five) [14]. Moreover, also in our study, nearly 60% of the STs that were identified were new. In contrast, the results obtained by Ikryannikova *et al.* conflict with ours and those of Bom *et al.*, as for 12 genovar E isolates, five MLVA-3 types but only two STs were described [2].

According to HGDI values, MLVA-3 showed the lowest discriminatory power (0.622), in contrast to other studies that found a better HGDI for genovar E, ranging from 0.782 to 0.902 [2], [5], [8], [29], while MLVA-5 and MLST presented similar HGDI values (0.913 and 0.860, respectively). Ideally, the HGDI should be 1.00, but in practice, it should be at least 0.90–0.95 for a typing system to be relatively optimal [30]. A combination of MLVA-5 and MLST resulted in the highest HGDI (0.949).

The application of the five VNTRs identified within genovar E permitted to describe 13 additional MLVA-5 types among non-E genovar specimens. However, to optimize the method, it would be interesting to look for new VNTRs for non-E genovar.

In summary, MLVA-5 has been developed for the genovar E *C. trachomatis* and used to type a large collection of French isolates and *C. trachomatis*-positive specimens belonging to genovar E and urogenital non-E genovars. Our findings show that MLVA can be used to discriminate between *C. trachomatis* isolates and can be directly applied to clinical specimens, thereby providing a high-resolution molecular epidemiological tool. Accordingly, the overall performance of the MLVA-5 assay would facilitate large-scale molecular epidemiological investigations of sexually transmitted *C. trachomatis* infections.

## Supporting Information

Table S1 **Characteristics of the 146** ***C. trachomatis*** **genovar E isolates and the 74 positive specimens used in this study.** ^a^Isolates and positive specimens are preceded by the letters I_E_ and S_E_, respectively. Specimens corresponding to isolates are indicated in parentheses. ^b^For the French isolates and specimens, the city is specified before the country. ^c^Isolates in bold were also typed by *ompA* sequencing, MLST and MLVA-3. ^d^Isolates and specimen belonging to the group II and the group III.
(DOC)

Table S2 **Characteristics of the 85 non-E genovar** ***C. trachomatis*** **-positive specimens and** ***C. trachomatis*** **reference strains.** ^a^Reference strains and positive specimens are preceded by the letters I and S, respectively.
(DOC)
