# Supplementary material for: MLVA Subtyping of Genovar E Chlamydia trachomatis Individualizes the Swedish Variant and Anorectal Isolates from Men who Have Sex with Men
Source: PLoS One. 2012 Feb 21;7(2):e31538. doi: 10.1371/journal.pone.0031538 (PMC3283677; doi:10.1371/journal.pone.0031538)
Supplement: Table S1 — Characteristics of the 146 C. trachomatis genovar E isolates and the 74 positive specimens used in this study. aIsolates and positive specimens are preceded by the letters IE and SE, respectively. Specimens corresponding to isolates are indicated in parentheses. bFor the French isolates and specimens, the city is specified before the country. cIsolates in bold were also typed by ompA sequencing, MLST and MLVA-3. dIsolates and specimen belonging to the group II and the group III. (DOC) [file pone.0031538.s001.doc]

Table S1.

| **Isolate or specimen**^a^ | **Sexe** | **Origin** | | **Date of isolation** | **MLVA-5 type** |
| --- | --- | --- | --- | --- | --- |
|  |  | **Country**^b^ | **Source** |  |  |
| ***Group I: urogenital clincial isolates and corresponding specimens when they were studied*** | | | | | |
| **I_E_refE/Bour**^c^ |  |  |  |  | 02 |
| **I_E_39** | M | Bordeaux, France | urethra | 08/12/1987 | 02 |
| **I_E_42** | M | Bordeaux, France | urethra | 18/11/1988 | 21 |
| I_E_56 | M | Bordeaux, France | urethra | 24/07/1984 | 02 |
| I_E_77 | F | Bordeaux, France | cervix | 31/03/1990 | 02 |
| I_E_121 | F | Bordeaux, France | peritoneal fluid | 25/10/1990 | 11 |
| I_E_128 | F | Bordeaux, France | intrauterine device | 29/11/1990 | 19 |
| I_E_174 | F | Bordeaux, France | cervix | 12/03/1991 | 11 |
| I_E_214 | F | Bordeaux, France | cervix | 16/09/1991 | 11 |
| **I_E_248** | M | Bordeaux, France | urethra | 09/01/1992 | 02 |
| I_E_266 | F | Bordeaux, France | cervix | 20/03/1992 | 02 |
| I_E_286 | F | Bordeaux, France | cervix | 12/05/1992 | 02 |
| I_E_296 | M | Bordeaux, France | urethra | 27/05/1992 | 11 |
| I_E_310 | F | Bordeaux, France | peritoneal fluid | 07/07/1992 | 02 |
| **I_E_315** | M | Bordeaux, France | urethra | 12/08/1992 | 22 |
| **I_E_316** | F | Bordeaux, France | cervix | 13/08/1992 | 22 |
| I_E_361 | F | Bordeaux, France | cervix | 11/01/1993 | 13 |
| **I_E_362** | F | Bordeaux, France | urethra | 15/01/1993 | 16 |
| I_E_367 | F | Bordeaux, France | cervix | 29/01/1993 | 06 |
| **I_E_374** | F | Bordeaux, France | cervix | 10/03/1993 | 10 |
| I_E_375 | F | Bordeaux, France | cervix | 04/11/1992 | 02 |
| I_E_377 | F | Bordeaux, France | cervix | 25/01/1993 | 13 |
| I_E_378 | F | Bordeaux, France | cervix | 17/02/1993 | 11 |
| I_E_385 | M | Bordeaux, France | urethra | 12/02/1993 | 13 |
| **I_E_386** | F | Bordeaux, France | cervix | 11/11/1992 | 11 |
| I_E_391 | F | Bordeaux, France | cervix | 26/11/1992 | 02 |
| I_E_395 | M | Bordeaux, France | urethra | 08/06/1993 | 11 |
| I_E_397 | F | Bordeaux, France | cervix | 26/05/1993 | 11 |
| I_E_400 | F | Bordeaux, France | cervix | 19/05/1993 | 11 |
| I_E_405 | F | Bordeaux, France | cervix | 05/06/1993 | 13 |
| I_E_409 | M | Bordeaux, France | urethra | 12/08/1993 | 01 |
| I_E_413 | M | Bordeaux, France | urethra | 29/09/1993 | 13 |
| I_E_427 | F | Bordeaux, France | cervix | 05/10/1993 | 02 |
| I_E_437 | F | Bordeaux, France | cervix | 18/11/1993 | 06 |
| I_E_442 | F | Bordeaux, France | cervix | 11/08/1993 | 11 |
| I_E_454 | M | Bordeaux, France | urethra | 09/03/1994 | 13 |
| I_E_457 | F | Bordeaux, France | cervix | 10/03/1994 | 11 |
| I_E_461 | F | Bordeaux, France | cervix | 04/01/1994 | 13 |
| **I_E_466** | F | Bordeaux, France | cervix | 03/01/1994 | 18 |
| I_E_469 | F | Bordeaux, France | cervix | 10/12/1993 | 13 |
| I_E_472 | F | Bordeaux, France | cervix | 08/04/1994 | 13 |
| I_E_474 | F | Bordeaux, France | peritoneal fluid | 19/04/1994 | 11 |
| I_E_479 | M | Bordeaux, France | urethra | 24/05/1994 | 11 |
| I_E_489 | F | Bordeaux, France | cervix | 10/04/1994 | 11 |
| I_E_499 | F | Bordeaux, France | cervix | 17/06/1994 | 11 |
| I_E_504 | F | Bordeaux, France | cervix | 19/07/1994 | 13 |
| **I_E_534** | F | Bordeaux, France | cervix | 30/08/1994 | 19 |
| I_E_535 | F | Bordeaux, France | cervix | 03/12/1994 | 11 |
| I_E_542 | F | Bordeaux, France | cervix | 12/04/1994 | 13 |
| I_E_555 | F | Bordeaux, France | cervix | 10/02/1995 | 11 |
| I_E_570 | M | Bordeaux, France | urethra | 02/03/1995 | 13 |
| I_E_574 | M | Bordeaux, France | urethra | 25/07/1995 | 02 |
| I_E_585 | F | Bordeaux, France | cervix | 23/09/1995 | 19 |
| **I_E_629** | F | Bordeaux, France | cervix | 10/01/1996 | 13 |
| I_E_632 | F | Bordeaux, France | cervix | 20/02/1996 | 11 |
| I_E_637 | F | Bordeaux, France | cervix | 27/05/1996 | 13 |
| **I_E_638** | F | Bordeaux, France | peritoneal fluid | 04/06/1996 | 04 |
| I_E_651 | F | Bordeaux, France | ovary | 12/06/1996 | 01 |
| I_E_652 | F | Bordeaux, France | cervix | 16/06/1996 | 05 |
| **I_E_662** | F | Bordeaux, France | cervix | 05/03/1997 | 10 |
| **I_E_663** | F | Bordeaux, France | cervix | 14/03/1997 | 13 |
| **I_E_665** | F | Bordeaux, France | cervix | 27/03/1997 | 17 |
| **I_E_774** | M | Bordeaux, France | urethra | 18/11/2002 | 09 |
| I_E_780 | F | Bordeaux, France | cervix | 09/03/2003 | 13 |
| **I_E_783** | M | Bordeaux, France | urethra | 09/04/2003 | 02 |
| I_E_798 | F | Bordeaux, France | cervix | 02/08/2004 | 11 |
| **I_E_802** (S_E_10163) | F | Bordeaux, France | cervix | 24/09/2004 | 13 |
| I_E_809 | F | Bordeaux, France | cervix | 11/11/2004 | 11 |
| I_E_811 | F | Bordeaux, France | cervix | 09/12/2004 | 19 |
| I_E_823 | F | Bordeaux, France | cervix | 11/05/2005 | 11 |
| **I_E_828** | F | Bordeaux, France | peritoneal fluid | 07/06/2005 | 11 |
| I_E_842 | F | Bordeaux, France | cervix | 16/03/2006 | 11 |
| I_E_856 (S_E_13096) | M | Bordeaux, France | urethra | 15/09/2006 | 13 |
| I_E_857 | F | Bordeaux, France | cervix | 20/09/2006 | 05 |
| **I_E_858** (S_E_13203) | F | Bordeaux, France | cervix | 29/09/2006 | 10 |
| **I_E_864** (S_E_13737) | F | Bordeaux, France | cervix | 11/12/2006 | 13 |
| I_E_870 | F | Bordeaux, France | cervix | 27/03/2007 | 13 |
| I_E_879 | F | Bordeaux, France | cervix | 23/04/2007 | 13 |
| I_E_882 | F | Bordeaux, France | cervix | 01/06/2007 | 11 |
| I_E_886 | F | Bordeaux, France | cervix | 25/06/2007 | 13 |
| I_E_887 | F | Bordeaux, France | cervix | 02/07/2007 | 05 |
| I_E_897 | M | Bordeaux, France | urethra | 29/10/2007 | 02 |
| I_E_900 (S_E_16493) | F | Bordeaux, France | cervix | 14/11/2007 | 02 |
| **I_E_901** | F | Bordeaux, France | cervix | 29/11/2007 | 09 |
| I_E_902 | F | Bordeaux, France | cervix | 02/12/2007 | 20 |
| I_E_910 (S_E_16877) | F | Bordeaux, France | cervix | 06/01/2008 | 02 |
| I_E_919 (S_E_17349) | F | Bordeaux, France | cervix | 20/02/2008 | 02 |
| I_E_921 (S_E_17350) | F | Bordeaux, France | cervix | 20/02/2008 | 08 |
| I_E_924 | F | Bordeaux, France | cervix | 23/03/2008 | 02 |
| I_E_925 | F | Bordeaux, France | cervix | 27/03/2008 | 02 |
| I_E_926 | F | Bordeaux, France | cervix | 01/04/2008 | 13 |
| I_E_929 | F | Bordeaux, France | cervix | 09/05/2008 | 07 |
| I_E_932 | M | Bordeaux, France | urethra | 04/06/2008 | 02 |
| I_E_948 | F | Bordeaux, France | cervix | 05/11/2008 | 11 |
| I_E_949 | M | Bordeaux, France | urethra | 18/11/2008 | 02 |
| I_E_952 | M | Bordeaux, France | urethra | 10/12/2008 | 02 |
| **I_E_957** | M | Bordeaux, France | bronchial aspiration | 19/01/2009 | 02 |
| I_E_963 (S_E_20643) | F | Bordeaux, France | cervix | 21/03/2009 | 08 |
| I_E_969 | F | Bordeaux, France | cervix | 11/05/2009 | 13 |
| I_E_973 (S_E_21369) | F | Bordeaux, France | cervix | 03/06/2009 | 13 |
| I_E_980 (S_E_21712) | F | Bordeaux, France | cervix | 14/07/2008 | 13 |
| I_E_984 | F | Bordeaux, France | cervix | 29/07/2009 | 13 |
| I_E_985 (S_E_21892) | F | Bordeaux, France | cervix | 02/08/2009 | 02 |
| ***Group II: concomitant urogenital isolates and specimens*** | | | | | |
| I_E_239^d^, **I_E_240** | F | Bordeaux, France | cervix, cervix | 13/12/1991 | 13 |
| I_E_263, I_E_264, I_E_265 | F | Bordeaux, France | endometrium, cervix, cervix | 16/03/1992 | 02 |
| **I_E_357^d^**, **I_E_358**,  I_E_359 | F | Bordeaux, France | cervix, peritoneal fluid, endometrium | 28/12/1992 | 02 |
| **I_E_392**, I_E_393 | F | Bordeaux, France | cervix, peritoneal fluid | 24/04/1993 | 15 |
| I_E_547, I_E_548 | F | Bordeaux, France | cervix, urethra | 10/05/1995 | 02 |
| I_E_641, I_E_642 | F | Bordeaux, France | cervix, endometrium | 28/06/1996 | 13 |
| **I_E_874** (S_E_14705),  **I_E_875** (S_E_14706) | F | Bordeaux, France | peritoneal fluid,  cervix | 30/03/2007 | 19 |
| S_E_23628, S_E_23629 | F | Bordeaux, France | cervix, urine | 20/01/2010 | 23 |
| S_E_23831, S_E_23832 | F | Bordeaux, France | cervix, urine | 05/02/2010 | 11 |
| S_E_23965, S_E_23968 | F | Bordeaux, France | cervix, urine | 12/02/2010 | 11 |
| S_E_24328, S_E_24329 | F | Bordeaux, France | cervix, urine | 09/03/2010 | 13 |
| S_E_24520, S_E_24521 | F | Bordeaux, France | cervix, urine | 23/03/2010 | 11 |
| S_E_24550^d^, S_E_24551  S_E_25654 | F | Bordeaux, France | cervix, urine  cervix | 25/03/2010  28/06/2010 | 11 |
| S_E_24904, S_E_24905 | M | Bordeaux, France | urine, urethra | 21/04/2010 | 24 |
| S_E_25417, S_E_25418 | M | Bordeaux, France | urine, urethra | 04/06/2010 | 13 |
| S_E_26196, S_E_26197 | M | Bordeaux, France | urine, urethra | 16/08/2010 | 13 |
| S_E_23720, S_E_23721 | F | Bordeaux, France | cervix, urine | 27/01/2010 | 01 |
| ***Group III: sequential urogenital isolates and specimens*** | | | | | |
| I_E_155, I_E_191 | F | Bordeaux, France | cervix, cervix | 19/11/1990, 07/04/1991 | 13 |
| I_E_205, I_E_272 | M | Bordeaux, France | urethra, urethra | 07/05/1991, 29/04/1992 | 03 |
| I_E_238, I_E_239^d^ | F | Bordeaux, France | cervix, cervix | 15/11/1991, 13/12/1991 | 13 |
| I_E_279, I_E_336 | F | Bordeaux, France | cervix, cervix | 02/06/1992, 15/12/1992 | 01 |
| I_E_325, I_E_527 | F | Bordeaux, France | cervix, cervix | 01/08/1992, 23/12/1994 | 02 |
| **I_E_344**, **I_E_488** | F | Bordeaux, France | cervix, cervix | 08/10/1992, 28/03/1994 | 11 |
| **I_E_357^d^**,  **I_E_600** | F | Bordeaux, France | cervix,  cervix | 28/12/1992,  03/12/1995 | 02  13 |
| I_E_792, I_E_800 | F | Bordeaux, France | cervix, cervix | 11/06/2004, 31/08/2004 | 11 |
| **I_E_863** (S_E_13681),  **I_E_918** (S_E_17268) | F | Bordeaux, France | cervix,  cervix | 04/12/2006,  12/02/2008 | 02 |
| I_E_909, I_E_913 (S_E_17097) | F | Bordeaux, France | cervix, cervix | 03/01/2008, 24/01/2008 | 13 |
| I_E_983, I**_E_986** (S_E_21582) | F | Bordeaux, France | cervix, cervix | 18/06/2009, 25/06/2009 | 12 |
| S_E_24550^d^, S_E_25654 | F | Bordeaux, France | cervix, cervix | 25/03/2010, 28/06/2010 | 11 |
| ***Group IV: urogenital positive specimens before and after treatment*** | | | | | |
| S_E_14758, S_E_15218 | F | Bordeaux, France | cervix, cervix | 04/04/2007, 01/06/2007 | 11 |
| S_E_24132, S_E_24848 | M | Bordeaux, France | urine, urine | 25/02/2010, 15/04/210 | 13 |
| S_E_24326, S_E_25165 | F | Bordeaux, France | cervix, cervix | 09/03/2010, 17/05/2010 | 02 |
| S_E_26549, S_E_18350 | F | Bordeaux, France | cervix, cervix | 10/09/2010, 09/11/2010 | 13 |
| S_E_18933, S_E_23785 | F | Bordeaux, France | cervix, cervix | 17/11/2010, 17/01/2011 | 25 |
| ***Group V: Swedish nvCT isolate and specimens*** | | | | | |
| I_E_871 | F | Bordeaux, France | cervix | 26/01/2007 | 14 |
| S_E_734 | M | Sweden | urine | 02/02/2009 | 14 |
| S_E_741 | M | Sweden | urine | 02/02/2009 | 14 |
| S_E_809 | M | Sweden | urine | 03/02/2009 | 14 |
| S_E_851 | F | Sweden | urine + cervix | 04/02/2009 | 14 |
| S_E_855 | F | Sweden | urine + cervix | 05/02/2009 | 14 |
| S_E_905 | F | Sweden | urine + cervix | 04/02/2009 | 14 |
| S_E_967 | F | Sweden | urine + cervix | 09/02/2009 | 14 |
| S_E_993 | M | Sweden | urine | 11/02/2009 | 14 |
| S_E_1010 | F | Sweden | urine + cervix | 12/02/2009 | 14 |
| S_E_1030 | F | Sweden | urine + cervix | 12/02/2009 | 14 |
| S_E_1221 | M | Sweden | urine | 30/01/2009 | 14 |
| S_E_1560 | M | Sweden | urine | 06/02/2009 | 14 |
| S_E_1664 | M | Sweden | urine | 09/02/2009 | 14 |
| S_E_1803 | M | Sweden | urine | 11/02/2009 | 14 |
| S_E_1965 | M | Sweden | urine | 16/02/2009 | 14 |
| S_E_2115 | M | Sweden | urine | 18/02/2009 | 14 |
| S_E_2667 | M | Sweden | urine | 02/03/2009 | 14 |
| S_E_2776 | M | Sweden | urine | 04/03/2009 | 14 |
| S_E_2978 | M | Sweden | urine | 10/03/2009 | 14 |
| S_E_3143 | M | Sweden | urine | 12/03/2009 | 14 |
| ***Group VI : anorectal isolates ans specimens*** | | | | | |
| **I_E_CV54** | M | Paris, France | anus/rectum | 01/06/2005 | 21 |
| **I_E_CV319** | M | Paris, France | anus/rectum | 06/01/2008 | 21 |
| **I_E_CV329** | M | Paris, France | anus/rectum | 15/03/2008 | 21 |
| **I_E_SL52** | M | Paris, France | anus/rectum | 17/08/2005 | 21 |
| **I_E_SL71** | M | Paris, France | anus/rectum | 19/06/2006 | 21 |
| **I_E_SL140** | M | Paris, France | anus/rectum | 20/12/2007 | 21 |
| S_E_26266 | M | Paris, France | anus/rectum | 19/08/2010 | 21 |
| S_E_26512 | M | Paris, France | anus/rectum | 10/09/2010 | 21 |
| S_E_26734 | M | Paris, France | anus/rectum | 28/09/2010 | 13 |
| S_E_26561 | M | Bordeaux, France | anus/rectum | 14/09/2010 | 21 |
| S_E_26838 | F | Bordeaux, France | anus/rectum | 05/10/2010 | 13 |
