# Supplementary material for: MLVA Subtyping of Genovar E Chlamydia trachomatis Individualizes the Swedish Variant and Anorectal Isolates from Men who Have Sex with Men
Source: PLoS One. 2012 Feb 21;7(2):e31538. doi: 10.1371/journal.pone.0031538 (PMC3283677; doi:10.1371/journal.pone.0031538)
Supplement: Table S2 — Characteristics of the 85 non-E genovar C. trachomatis -positive specimens and C. trachomatis reference strains. aReference strains and positive specimens are preceded by the letters I and S, respectively. (DOC) [file pone.0031538.s002.doc]

Table S2.

| **Reference strain**  **or specimen**^a^ | **Sexe** | **Origin** | | **Date of isolation** | **Genovar** | **MLVA-5 type** |
| --- | --- | --- | --- | --- | --- | --- |
|  |  | **City, Country** | **Source** |  |  |  |
| I_D_ref UW-3/Cx |  |  |  |  | D | 27 |
| S_D_524 | F | Bordeaux, France | cervix | 03/11/1994 | D | 26 |
| S_D_530 | F | Bordeaux, France | cervix | 03/11/1994 | D | 09 |
| S_D_612 | F | Bordeaux, France | cervix | 01/03/1996 | D | 32 |
| S_D_619 | F | Bordeaux, France | cervix | 01/04/1996 | D | 27 |
| S_D_20576 | F | Bordeaux, France | cervix | 02/02/2009 | D | 08 |
| I_Da_ref TW-448/Cx |  |  |  |  | Da | 17 |
| S_Da_761 | M | Bordeaux, France | urethra | 09/04/2002 | Da | 08 |
| S_Da_8769 | F | Bordeaux, France | cervix | 14/03/2003 | Da | 09 |
| S_Da_11033 | F | Bordeaux, France | cervix | 20/03/2005 | Da | 09 |
| S_Da_11389 | M | Bordeaux, France | urethra | 17/05/2005 | Da | 31 |
| S_Da_11689 | M | Bordeaux, France | urethra | 18/05/2005 | Da | 09 |
| S_Da_12592 | M | Bordeaux, France | anus/rectum | 28/06/2006 | Da | 09 |
| S_Da_17909 | F | Bordeaux, France | cervix | 02/05/2008 | Da | 17 |
| S_Da_18913 | M | Bordeaux, France | urethra | 23/09/2008 | Da | 32 |
| S_Da_K10 | M | Paris, France | anus/rectum | 02/08/2004 | Da | 08 |
| S_Da_CV450 | M | Paris, France | anus/rectum | 01/07/2008 | Da | 09 |
| S_Da_BX1 | M | Bordeaux, France | anus/rectum | 18/02/2010 | Da | 26 |
| S_Da_F503 | M | Paris, France | anus/rectum | 15/03/2010 | Da | 26 |
| S_Da_F513 | M | Paris, France | anus/rectum | 10/05/2010 | Da | 26 |
| S_Da_CB16 | M | Paris, France | anus/rectum | 01/06/2010 | Da | 27 |
| S_Da_TG12 | M | Tourcoing, France | anus/rectum | 11/06/2010 | Da | 26 |
| S_Da_CB20 | M | Paris, France | anus /rectum | 28/06/2010 | Da | 09 |
| S_Da_F521 | M | Paris, France | anus /rectum | 30/07/2010 | Da | 26 |
| S_Da_CV597 | M | Paris, France | anus /rectum | 07/08/2010 | Da | 28 |
| S_Da_SL234 | M | Paris, France | anus /rectum | 17/08/2010 | Da | 28 |
| S_Da_LYO2 | M | Lyon, France | anus /rectum | 29/09/2010 | Da | 28 |
| S_Da_CV628 | M | Paris, France | anus /rectum | 27/10/2010 | Da | 29 |
| I_F_ref Ic-Cal-3 |  |  |  |  | F | 09 |
| S_F_23787 | F | Bordeaux, France | urine | 01/07/2010 | F | 08 |
| S_F_24476 | F | Bordeaux, France | cervix | 19/03/2010 | F | 09 |
| S_F_24692 | F | Bordeaux, France | urine | 01/04/2010 | F | 17 |
| S_F_26122 | F | Bordeaux, France | cervix | 10/08/2010 | F | 08 |
| S_F_26844 | F | Bordeaux, France | cervix | 06/10/2010 | F | 38 |
| S_F_18611 | F | Bordeaux, France | cervix | 12/11/2010 | F | 09 |
| S_F_23512 | M | Paris, France | anus/rectum | 06/01/2010 | F | 11 |
| S_F_CV648 | M | Paris, France | anus/rectum | 20/12/2010 | F | 11 |
| I_G_ref UW-57/Cx |  |  |  |  | G | 27 |
| S_G_23457 | F | Bordeaux, France | cervix | 07/01/2010 | G | 32 |
| S_G_24816 | F | Bordeaux, France | cervix | 14/04/2010 | G | 32 |
| S_G_25258 | F | Bordeaux, France | cervix | 21/05/2010 | G | 32 |
| S_G_26301 | F | Bordeaux, France | cervix | 24/08/2010 | G | 32 |
| S_G_26745 | M | Bordeaux, France | urine | 28/09/2010 | G | 11 |
| S_G_22203 | F | Bordeaux, France | cervix | 29/12/2010 | G | 09 |
| S_G_F495 | M | Paris, France | anus/rectum | 08/01/2010 | G | 36 |
| S_G_CV607 | M | Paris, France | anus/rectum | 16/09/2010 | G | 26 |
| S_G_4711 | M | Monptellier, France | anus/rectum | 01/10/2010 | G | 26 |
| S_G_CV633 | M | Paris, France | anus/rectum | 11/11/2010 | G | 09 |
| S_G_CV639 | M | Paris, France | anus/rectum | 30/11/2010 | G | 37 |
| S_G_23514 | M | Paris, France | anus/rectum | 07/01/2010 | G | 26 |
| S_G_23903 | M | Tourcoing, France | anus/rectum | 09/02/2010 | G | 36 |
| S_G_26462 | M | Bordeaux, France | anus/rectum | 06/09/2010 | G | 26 |
| S_G_19250 | F | Bordeaux, France | cervix | 02/11/2008 | G | 08 |
| S_G_17356 | F | Bordeaux, France | cervix | 21/02/2008 | G | 11 |
| S_G_CV601 | M | Paris, France | anus/rectum | 28/08/2010 | G | 28 |
| I_H_ref UW-43/Cx |  |  |  |  | H | 27 |
| S_H_25875 | F | Bordeaux, France | cervix | 19/07/2010 | H | 32 |
| S_H_20197 | F | Bordeaux, France | cervix | 06/02/2009 | H | 32 |
| S_H_21447 | F | Bordeaux, France | cervix | 11/06/2009 | H | 17 |
| I_I_ref UW-12/Ur |  |  |  |  | I | 32 |
| S_I_25783 | F | Bordeaux, France | cervix | 08/07/2010 | I | 32 |
| S_I_22200 | F | Bordeaux, France | cervix | 02/09/2009 | I | 32 |
| S_I_22592 | F | Bordeaux, France | cervix | 09/10/2009 | I | 32 |
| S_I_22859 | F | Bordeaux, France | cervix | 05/11/2009 | I | 17 |
| S_I_22890 | F | Bordeaux, France | cervix | 09/11/2009 | I | 32 |
| S_I_22906 | F | Bordeaux, France | cervix | 09/11/2009 | I | 17 |
| I_Ia_ref UW-202/Cx |  |  |  |  | Ia | 17 |
| S_Ia_23549 | M | Bordeaux, France | urine | 14/01/2010 | Ia | 17 |
| S_Ia_24206 | F | Bordeaux, France | cervix | 02/03/2010 | Ia | 27 |
| S_Ia_24870 | F | Bordeaux, France | cervix | 16/04/2010 | Ia | 27 |
| S_Ia_20428 | F | Bordeaux, France | cervix | 03/12/2010 | Ia | 17 |
| S_Ia_26719 | F | Bordeaux, France | cervix | 27/09/2010 | Ia | 17 |
| S_Ia_22142 | F | Bordeaux, France | cervix | 28/08/2009 | Ia | 17 |
| S_Ia_26797 | F | Bordeaux, France | cervix | 01/10/2010 | Ia | 17 |
| I_J_ref UW-36/Cx |  |  |  |  | J | 32 |
| S_J_23661 | F | Bordeaux, France | cervix | 22/01/2010 | J | 27 |
| S_J_26206 | F | Bordeaux, France | cervix | 17/08/2010 | J | 17 |
| S_J_26223 | M | Bordeaux, France | urine | 18/08/2010 | J | 33 |
| S_J_26889 | F | Bordeaux, France | cervix | 08/10/2010 | J | 17 |
| S_J_16887 | F | Bordeaux, France | cervix | 20/10/2010 | J | 17 |
| S_J_21148 | F | Bordeaux, France | cervix | 12/05/2009 | J | 17 |
| S_J_21739 | F | Bordeaux, France | cervix | 17/07/2009 | J | 15 |
| S_J_22510 | F | Bordeaux, France | cervix | 01/10/2009 | J | 17 |
| S_J_CV533 | M | Paris, France | anus/rectum | 21/01/2010 | J | 30 |
| S_J_F505 | M | Paris, France | anus/rectum | 02/04/2010 | J | 30 |
| S_J_CV609 | M | Paris, France | anus/rectum | 27/09/2010 | J | 30 |
| S_J_CB37 | M | Paris, France | anus/rectum | 09/11/2010 | J | 31 |
| S_J_TG23 | M | Tourcoing, France | anus/rectum | 22/12/2010 | J | 34 |
| S_J_CV599 | M | Bordeaux, France | anus/rectum | 23/08/2010 | J | 33 |
| S_J_CV605 | M | Paris, France | anus/rectum | 15/09/2010 | J | 35 |
| I_K_ref UW-31/Cx |  |  |  |  | K | 27 |
| S_K_23397 | F | Bordeaux, France | cervix | 04/01/2010 | K | 32 |
| S_k_25192 | F | Bordeaux, France | cervix | 19/05/2010 | K | 32 |
| S_K_23515 | M | Bordeaux, France | urine | 12/01/2011 | K | 32 |
| S_K_23516 | F | Bordeaux, France | cervix | 12/01/2011 | K | 32 |
